# Identification of a novel CG307 sub-clade in third-generation-cephalosporin-resistant *Klebsiella pneumoniae* causing invasive infections in the USA

**DOI:** 10.1099/mgen.0.001201

**Published:** 2024-02-26

**Authors:** Selvalakshmi Selvaraj Anand, Chin-Ting Wu, Jordan Bremer, Micah Bhatti, Todd J. Treangen, Awdhesh Kalia, Samuel A. Shelburne, William C. Shropshire

**Affiliations:** ^1^​ Graduate Program in Diagnostic Genetics and Genomics, School of Health Professions, MD Anderson Cancer Center, University of Texas, Houston, TX, USA; ^2^​ Department of Infectious Diseases, Infection Control, and Employee Health, MD Anderson Cancer Center, University of Texas, Houston, TX, USA; ^3^​ Department of Laboratory Medicine, MD Anderson Cancer Center, University of Texas, Houston, TX, USA; ^4^​ Department of Computer Science, Rice University, Houston, TX, USA; ^5^​ Department of Genomic Medicine, MD Anderson Cancer Center, University of Texas, Houston, TX, USA

**Keywords:** accessory genome, clonal group 307, multidrug-resistant *K. pneumoniae* surveillance, nosocomial transmission, third-generation-cephalosporin-resistant *Klebsiella pneumoniae*

## Abstract

Despite the notable clinical impact, recent molecular epidemiology regarding third-generation-cephalosporin-resistant (3GC-R) *Klebsiella pneumoniae* in the USA remains limited. We performed whole-genome sequencing of 3GC-R *K. pneumoniae* bacteraemia isolates collected from March 2016 to May 2022 at a tertiary care cancer centre in Houston, TX, USA, using Illumina and Oxford Nanopore Technologies platforms. A comprehensive comparative genomic analysis was performed to dissect population structure, transmission dynamics and pan-genomic signatures of our 3GC-R *K*. *pneumoniae* population. Of the 178 3GC-R *K. pneumoniae* bacteraemias that occurred during our study time frame, we were able to analyse 153 (86 %) bacteraemia isolates, 126 initial and 27 recurrent isolates. While isolates belonging to the widely prevalent clonal group (CG) 258 were rarely observed, the predominant CG, 307, accounted for 37 (29 %) index isolates and displayed a significant correlation (Pearson correlation test *P* value=0.03) with the annual frequency of 3GC-R *K*. *pneumoniae* bacteraemia. Interestingly, only 11 % (4/37) of CG307 isolates belonged to the commonly detected ‘Texas-specific’ clade that has been observed in previous Texas-based *K. pneumoniae* antimicrobial-resistance surveillance studies. We identified nearly half of our CG307 isolates (*n*=18) belonged to a novel, monophyletic CG307 sub-clade characterized by the chromosomally encoded *bla*_SHV-205_ and unique accessory genome content. This CG307 sub-clade was detected in various regions of the USA, with genome sequences from 24 additional strains becoming recently available in the National Center for Biotechnology Information (NCBI) SRA database. Collectively, this study underscores the emergence and dissemination of a distinct CG307 sub-clade that is a prevalent cause of 3GC-R *K*. *pneumoniae* bacteraemia among cancer patients seen in Houston, TX, and has recently been isolated throughout the USA.

## Data Summary

Whole-genome-sequencing (WGS) data collected during this study period was submitted to the National Center for Biotechnology Information (NCBI) and can be accessed within BioProject PRJNA648389. WGS data from a previous study of carbapenem non-susceptible *Enterobacterales* can be accessed from BioProject PRJNA836696. Assembly information and accession numbers are provided in Table S1 (available with the online version of this article).

**Impact Statement**

Infections due to third-generation-cephalosporin-resistant (3GC-R) *Klebsiella pneumoniae* are considered among the most urgent public-health threats. However, molecular epidemiology studies on 3GC-R *K*. *pneumoniae* in the USA are limited. Our analysis indicates a preponderance of genetically diverse 3GC-R *K*. *pneumoniae* isolates harbouring the key antimicrobial-resistance (AMR) determinant *bla*_CTX-M-15_ at our institution. Importantly, however, we detected evidence of long-duration transmission of highly genetically related clonal group (CG) 307 and CG29 specific clusters at our institution. Interestingly, we rarely detected the pandemic CG258 lineage in our cohort and did not detect more than two genetically related CG258 isolates from this lineage. We found that approximately 50 % of our isolates from the most prevalent CG, 307, belonged to a novel CG307 clade that is highly divergent from a more commonly detected Texas-specific clade that has circulated in our region. We searched the National Center for Biotechnology Information (NCBI) SRA database using genomic markers of the novel CG307 clade and found evidence of this clade causing recent invasive infections in other locations across the USA. Our study highlights the shifting population dynamics of *K. pneumoniae* causing invasive infections and the necessity to continue AMR surveillance in order to identify emerging high-risk populations.

## Introduction

The necessity to address antimicrobial-resistant infections as a global public-health threat has become increasingly apparent during the 21st century. A recent study estimated that 4.95 million deaths were associated with antimicrobial-resistant infections worldwide, with *Klebsiella pneumoniae* infections being the third most-common pathogen associated with mortality [1]. *K. pneumoniae*, a member of the family *Enterobacterales*, is an opportunistic pathogen that has the capacity to develop resistance to multiple classes of antibiotics. A recent meta-analysis estimated about 33 % of nosocomial *K. pneumoniae* infections are caused by multidrug-resistant (MDR) strains, highlighting the impact of these pathogens in the healthcare setting [2].

MDR *K. pneumoniae* clonal populations can acquire β-lactamase-encoding genes such as extended-spectrum β-lactamase (ESBL) or AmpC-encoding genes via horizontal gene transfer (HGT), which results in third-generation-cephalosporin resistance. These HGT plasmid vectors can also harbour carbapenemase-encoding genes such as *bla*_KPC,_ which encodes the *K. pneumoniae* carbapenemase (KPC) that effectively hydrolyses most classes of β-lactams [3–12]. One of the most concerning MDR *K. pneumoniae* clonal populations is the pandemic group of strains known as clonal group (CG) 258, which includes sequence type 258 (ST258), ST512 and ST11, which are all closely related (i.e. the CG258 mean pairwise SNP distance is ~214) and strongly associated with worldwide *bla*_KPC_ dissemination [6, 13–17]. ST258 strains are the predominant MDR *K. pneumoniae* detected in the USA over the past two decades [5, 7, 16–19].

While ST258 prevalence has remained high in certain regions of the USA, there is growing evidence of an emergent CG307 lineage co-circulating with CG258 in the Houston, TX, region [7, 9, 20, 21]. When performing comparative genomics between the two lineages, a discerning factor is the strong association of the ESBL-encoding gene *bla*_CTX-M-15_ that is present in >90 % of CG307 isolates, whereas *bla*_CTX-M-15_ is detected in <10 % of CG258 isolates [9, 10, 20]. Recent molecular epidemiological investigations of CG307 isolates have identified two predominant CG307 clades with a strong phylogenetic signal that distinguishes isolates of Texas origin from those from the more globally disseminated clade [10, 20]. When comparing the two ST307 lineages, the Texas-specific clade has a stable chromosomal insertion of two IS*Ecp1-bla*_CTX-M-15_ transposition units, whereas the more globally disseminated CG307 lineage harbours *bla*_CTX-M-15_ primarily on large, multireplicon, F-type conjugative plasmids [8, 10, 20]. Furthermore, recent antimicrobial resistance (AMR) surveillance studies from Europe have highlighted how CG307 strains are the primary drivers of third-generation-cephalosporin-resistant (3GC-R) *K*. *pneumoniae* infections within hospitals, in part due to their strong association with *bla*_CTX-M-15_ carriage and transmission [22–24].

Given the high prevalence of CG307 in Houston and surrounding areas [7, 9, 20, 21, 25, 26], as well as a lack of current USA-based 3GC-R *K*. *pneumoniae* surveillance studies, we sought to use whole-genome-sequencing (WGS) to determine the molecular epidemiology of 3GC-R *K*. *pneumoniae* causing bacteraemia in cancer patients at our institution within a 5 year time frame. We found that CG307 strains were the most common CG detected, whereas the previously highly prevalent CG258 was rarely identified. Interestingly, 90 % of CG307 bacteraemias were caused by strains of the global clade; in particular, we identified that a previously unidentified, global sub-clade with distinct genomic signatures caused approximately 50 % of the CG307 bacteraemias at our institution. A search of publicly available WGS data identified isolates of this sub-clade recently collected from geographically diverse sites throughout the USA. These data expand upon the rapidly changing epidemiology of 3GC-R *K*. *pneumoniae*, including the increasing importance of emerging, diverse CG307 sub-clades.

## Methods

### Study design and sample collection

The study included all *K*. *pneumoniae* bacteraemias that occurred from March 1st 2016, chosen coincident with the date of implementation of the Epic Electronic Health Record system at the University of Texas MD Anderson Cancer Center (MDACC) in Houston, TX, to May 31st 2022. We defined an index *K. pneumoniae* bacteraemia isolate as the first occurrence of a positive blood culture, and a recurrent *K. pneumoniae* bacteraemia isolate as a positive blood culture that occurred at least 14 days from a previous *K. pneumoniae* bacteraemia isolate. Antibiotic-susceptibility testing of all *K. pneumoniae* bacteraemia isolates was performed by the MDACC microbiology laboratory using the Accelerate PhenoTest BC (Accelerate Diagnostics), ETEST (bioMérieux) and VITEK 2 (bioMérieux) systems with susceptibility interpretations based on Clinical and Laboratory Standards Institute (CLSI) guidelines [27]. *K. pneumoniae* bacteraemia isolates were considered as extended-spectrum-cephalosporin resistant (3GC-R) if they had a ceftriaxone (CRO) minimum inhibitory concentration (MIC) ≥4 µg ml^−1^ and/or a predicted ESBL phenotype as determined by the VITEK2 Advanced Expert system. Out of the 178 3GC-R *K*. *pneumoniae* causing bacteraemia during the study period, there were 91 % (161/178) 3GC-R *K*. *pneumoniae* isolates available for sequencing. Further information on sampling is included in the Results section.

### Short-read and long-read sequencing

3GC-R *K. pneumoniae* isolates were stocked in thioglycolate broth supplemented with 40 % (v/v) glycerol as part of an ongoing surveillance of bacteraemia isolates. MicroBank (MB) numbers are unique to patient infections and deidentified. Samples were plated onto trypticase soy agar (TSA with sheep blood). These plates were subsequently incubated overnight at 37 °C and 4 % CO_2_. After 12–24 h, a single colony was inoculated in autoclaved Miller’s LB broth and incubated at 37 °C for 2 h with mild agitation (225 r.p.m.). Two millilitres of the inoculated media were aliquoted into 15 ml Eppendorf tubes and spun down to pellet cells. Genomic DNA (gDNA) was extracted according to the protocol specified by the QIAGEN Blood and Cell Culture DNA kit (catalogue no. 69581).

3GC-R *K. pneumoniae* isolates were sequenced by Illumina NovaSeq 6000, as previously described [21]. There were 126 3GC-R *K. pneumoniae* isolates with 150 bp paired-end reads that passed quality control as assessed using fastqc v0.11.9 (https://github.com/s-andrews/FastQC). We checked fastqc output for passing the ‘per base sequence quality’ metric; additionally, all sequences passed National Center for Biotechnology Information (NCBI) internal quality checks. We performed long-read sequencing on nine isolates of interest that were part of transmission clusters to obtain their complete genomes using the Oxford Nanopore Technologies (ONT) MinION platform, as described previously [21]. Briefly, gDNA extracted for the purpose of short-read sequencing was used as input for ONT long-read sequencing. Library preparation was accomplished using the Rapid Sequencing kit 96 v10 (SQK-RBK110.96). The input gDNA was normalized to 50 ng to ensure even distributions of libraries across pooled samples. The prepared libraries were loaded onto the R9.4.1 flowcell (FLO-MIN106D) for sequencing with MinKNOW software to generate fast5 files. Guppy v6.4.6 basecaller was used to perform basecalling from the fast5 files to obtain fastq files using super high accuracy model (SUP).

### Short- and long-read sequencing data analysis

Quality assessed, paired-end 150 bp reads were assembled via SPAdes v3.15.5 [28] with default parameters and the inclusion of the isolate option. The quality assessment, assignment of STs, CGs and capsule type of isolates were assessed using Kleborate v2.2.0 with Kaptive v2.0.0 activated [29, 30]. One genome assembly had Kleborate quality control warnings due to ambiguous bases, genome size >7.5 or <4.5 Mbp, or N50 <10 kbp and was removed from analysis. Full Kleborate output is available in Table S2. The genome assemblies of 153 isolates included in our study were submitted to the Institute Pasteur for assigning dual barcodes based on multilevel single locus clustering and life identification numbers (LIN) using a curated 629 gene core-genome multilocus sequence typing scheme [31]. This recently developed typing strategy standardizes nomenclature in an effort to improve surveillance of high-risk antimicrobial-resistant *K. pneumoniae* pathogens. The COpy Number Variant quantifICation Tool (convict v1.0) was used to identify AMR from the ResFinder database (accessed 09-11-2021) and estimate gene copy numbers, as described previously (W. C. Shropshire, convict, GitHub: https://github.com/wshropshire/convict) [21]. convict as part of its pipeline employs the KmerResistance-2.0 bioinformatic tool that uses short-read *k*-mer alignment (KMA) to identify homologous AMR genes from redundant databases [32, 33]. The NCBI AMRFinderPlus command-line tool (v3.11.14) was used to confirm AMR gene identification form convict using the NCBI curated database (version 2023-04-17.1).

A short- and long-read assembly pipeline (W. C. Shropshire, flye_hybrid_assembly_pipeline, GitHub: https://github.com/wshropshire/flye_hybrid_assembly_pipeline) was used to close complete genomes of ONT-sequenced data, as described previously [21]. Incomplete assemblies were re-assembled using Unicycler v0.5.0 and manually curated for errors using short- and long-read pileups and visualizing with the integrated genome browser (IGV v2.14.1) [34]. The high-performance computing (HPC) cluster, Seadragon, that is hosted through MDACC was used to perform genomic analyses. Sequencing quality control, third-generation-cephalosporin-resistance gene determinants and antimicrobial-susceptibility testing data for isolates sequenced for this project, in addition to previous MDACC studies, are available in Table S1.

### Core-gene and genome analyses of 3GC-R *K. pneumoniae* isolates

Genomes from index isolates (*n*=126) were annotated using Prokka v1.14.5 and the annotation files in generalized feature format (GFF3) were used for the subsequent pan-genome analysis [35]. The GFF3 files from Prokka were used to make a core-gene alignment through Roary v3.13 using mafft v7.4 [36, 37]. The index 3GC-R *K. pneumoniae* core gene length was 3 720 547 bases. The pairwise SNP distances from the core-gene alignment were identified using snp-dists v0.8.4 (https://github.com/tseemann/snp-dists). iq-tree v2.0.6 was used to recreate a maximum-likelihood phylogeny using the core-gene alignment to determine the population structure [38]. Bootstrap analysis was performed using UFBoot approximation and SH-like approximate likelihood test with 1000 replicates though iq-tree v2.0.6 [38, 39]. The tree visualization was performed using the R package ggtree v3.9.1.

PopPUNK v2.6.0 was used with index 3GC-R *K. pneumoniae* draft assemblies (*n*=126) to identify potential cluster networks based on the core and accessory genome [40]. Following assignment, a core-SNP phylogeny for each PopPUNK group was performed with a complete genome reference isolate using snippy v4.6.0 (https://github.com/tseemann/snippy). The recombination regions were masked using Gubbins v2.3.4 [36] and the filtered alignment file was used as the input file to obtain a core-SNP maximum-likelihood phylogeny using iq-tree v2.0.6 [38]. The filtered polymorphic sites were used to generate pairwise SNP matrices to determine genetic relatedness within each specific PopPUNK group.

For CG307 population structure analysis, we performed a convenience sampling of publicly available CG307 isolates from diverse geographical locales and collection dates, specifically oversampling for CG307 isolates harbouring *bla*_SHV-205_ alleles. CG307 short-reads were downloaded from NCBI using the sratoolkit v2.10.9 through the fasterq-dump function (https://github.com/ncbi/sra-tools). NCBI SRA accession numbers and metadata for CG307 isolates are available in Table S3. A core-SNP phylogeny for the CG307 isolates was performed using Kp616 (GenBank accession no. GCA_003076555.1) as a reference isolate using snippy v4.6.0, as described in the paragraphs above. The Kp616 isolate was selected as a reference as it was previously used by Wyres *et al*. for comparing CG307 population structure in addition to the genome being closed, completely resolved and collected in 2009 [10]. The recombination regions were masked using Gubbins v2.3.4 and a core-SNP maximum-likelihood phylogeny using iq-tree v2.0.6 was performed, as mentioned elsewhere [38]. The per branch statistics output from Gubbins was analysed to look for regions of high recombination.

A Bayesian analysis of population structure was performed using the core-genome alignment through rhierBAPS v1.0.1 to identify CG307-specific clusters [41]. Bayesian dating of the nodes of the CG307 phylogenetic tree was performed using BactDating v1.1.0 (https://xavierdidelot.github.io/BactDating/). The recombination-free core-SNP phylogenetic tree output from Gubbins v2.3.4 was used to perform root-to-tip regression analysis prior to molecular dating (Fig. S1). The output timed tree was further annotated and visualized using R package ggtree v3.9.1.

### Accessory genome analysis

We used a subset of the accessory genome excluding low (<5 %) and high frequency (>95 %) genes for agglomerative hierarchical clustering of the gene presence/absence matrix from Roary to analyse the accessory genomes of CG307. Comparison of plasmid vectors with blast ring image generator (brig) was performed using the Proksee webserver [42]. Phage content in the genomes was characterized using the phaster webserver (https://phaster.ca/). Replicon types were identified using the ‘PlasmidFinder’ database (accessed 01-07-2023) with ABRicate v1.0.0 (https://github.com/tseemann/abricate). Data visualization was generated using R v4.0.4 packages or Geneious Prime software (2023.2.1).

### Statistical analysis

Time series data were assessed using the Mann–Kendall trend test. Comparisons between rates of *K. pneumoniae* bacteraemias by 6 month groups were performed using Student’s *t*-test. Chi-square tests were used to compare group proportions between *K. pneumoniae* bacteraemias. All statistics was computed on R v4.0.4 or Prism 9 with a type one error rate (α=0.05) used across all hypothesis testing.

## Results

### Identification of higher 3GC-R *K. pneumoniae* bacteraemia prevalence in the latter half of annual periods

**Fig. 1.**
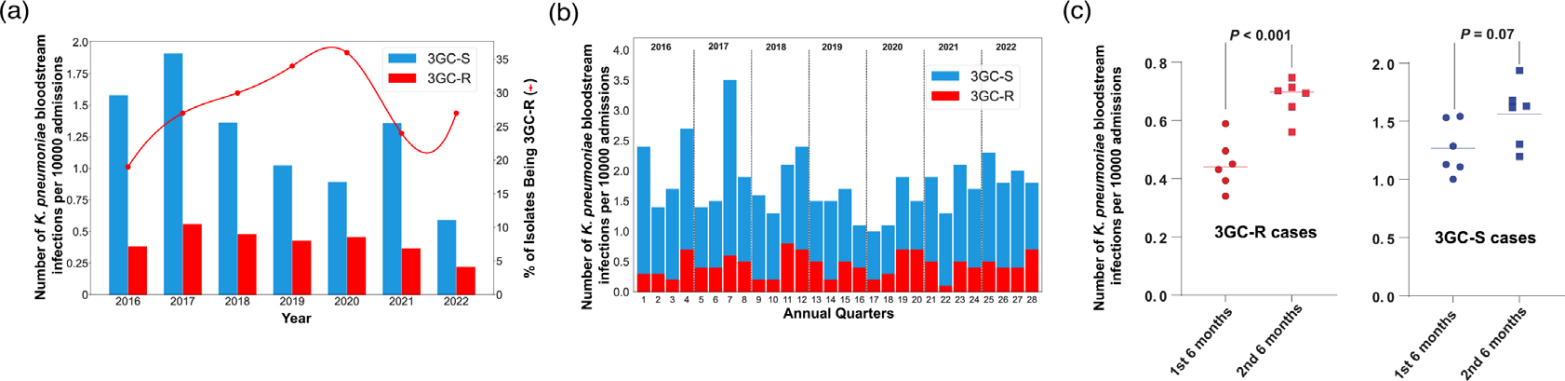
Epidemiology of *K. pneumoniae* bacteraemias. (**a**) Index *K. pneumoniae* bacteraemias by year. The *x*-axis shows the year, and the *y*-axis (left) shows the normalized number of bacteraemias, with blue bars representing the 3GC-S *K. pneumoniae,* and red bars representing the 3GC-R *K. pneumoniae* infections. The other *y*-axis (right) shows the proportion of 3GC-R *K. pneumoniae* isolates amongst total *K. pneumoniae* bacteraemias (red line). (**b**) *K. pneumoniae* bacteraemias stratified by quarters of the year. The *x*-axis represents quarters of the year, and the *y*-axis represents the normalized number of *K. pneumoniae* bacteraemias with 3GC-R *K. pneumoniae* (red) and 3GC-S *K. pneumoniae* (blue) labelled. (**c**) Comparison of 3GC-R and 3GC-S *K. pneumoniae* bacteraemia in the first half and the second half of the year. The red dots on the left represent the mean number of 3GC-R *K. pneumoniae* bacteraemia for each month (e.g. January, February, etc.), and the blue dots on the right represent the mean number of 3GC-S *K. pneumoniae* bacteraemias by month. Student’s *t*-test *P* values are labelled accordingly.

Out of 621 *K*. *pneumoniae* bacteraemias that occurred from March 2016 to May 2022, 89 % (555/621) were index infections (i.e. the first occurrence of a patient *K. pneumoniae* bacteraemia episode). There were 49 (9 %) patients with index bacteraemias that had subsequently one or more recurrent bacteraemia episodes (i.e. bacteraemia occurring ≥14 days following an index bacteraemia episode). When stratifying by number of recurrent episodes, 78 % (38/49) of patients had one recurrent episode, 14 % (7/49) had two recurrent episodes, 4 % (2/49) had three recurrent episodes and 4 % (2/49) had four recurrent episodes. 3GC-R *K. pneumoniae* infections accounted for 29 % (178/621) of *K. pneumoniae* bacteraemias, of which 78 % (138/178) were index infections. The proportion of patients with index bacteraemias who had recurrent episodes was significantly higher for 3GC-R *K. pneumoniae* (19 %, 26/138) compared to third-generation-cephalosporin-susceptible (3GC-S) *K. pneumoniae* infections (6 %, 23/417, χ^2^ *P* value <0.0001). When normalized by the number of admissions to account for fluctuations in patient volume, the rates of index 3GC-R *K. pneumoniae* bacteraemias were not significantly different over the course of the study (Mann–Kendall trend test, *P* value >0.05) (Fig. 1a). Similar to our recent study of 3GC-R *Escherichia coli* bacteraemia [43], 3GC-R *K. pneumoniae* bacteraemia prevalence was significantly higher in the last 6 months of the year relative to the first 6 months (0.67 vs 0.45 bacteraemias per month per 1000 patient admissions, respectively; Student’s *t*-test, *P* value <0.001) (Fig. 1b, c). No statistically significant difference in 3GC-S *K. pneumoniae* bacteraemias stratified by time of year was observed (1.6 vs 1.3 bacteraemias per month per 1000 patient admissions in the second half of year vs the first half of year, respectively; Students *t*-test, *P* value=0.07; Fig. 1c). These data indicate that for the past several years the rates of 3GC-R *K. pneumoniae* bacteraemia have not been significantly changing and that environmental factors may contribute to 3GC-R *K. pneumoniae* infections.

### CG307 strains caused more 3GC-R *K. pneumoniae* bacteraemias during the study time frame compared to other 3GC-R *K. pneumoniae* genotypes

**Fig. 2.**
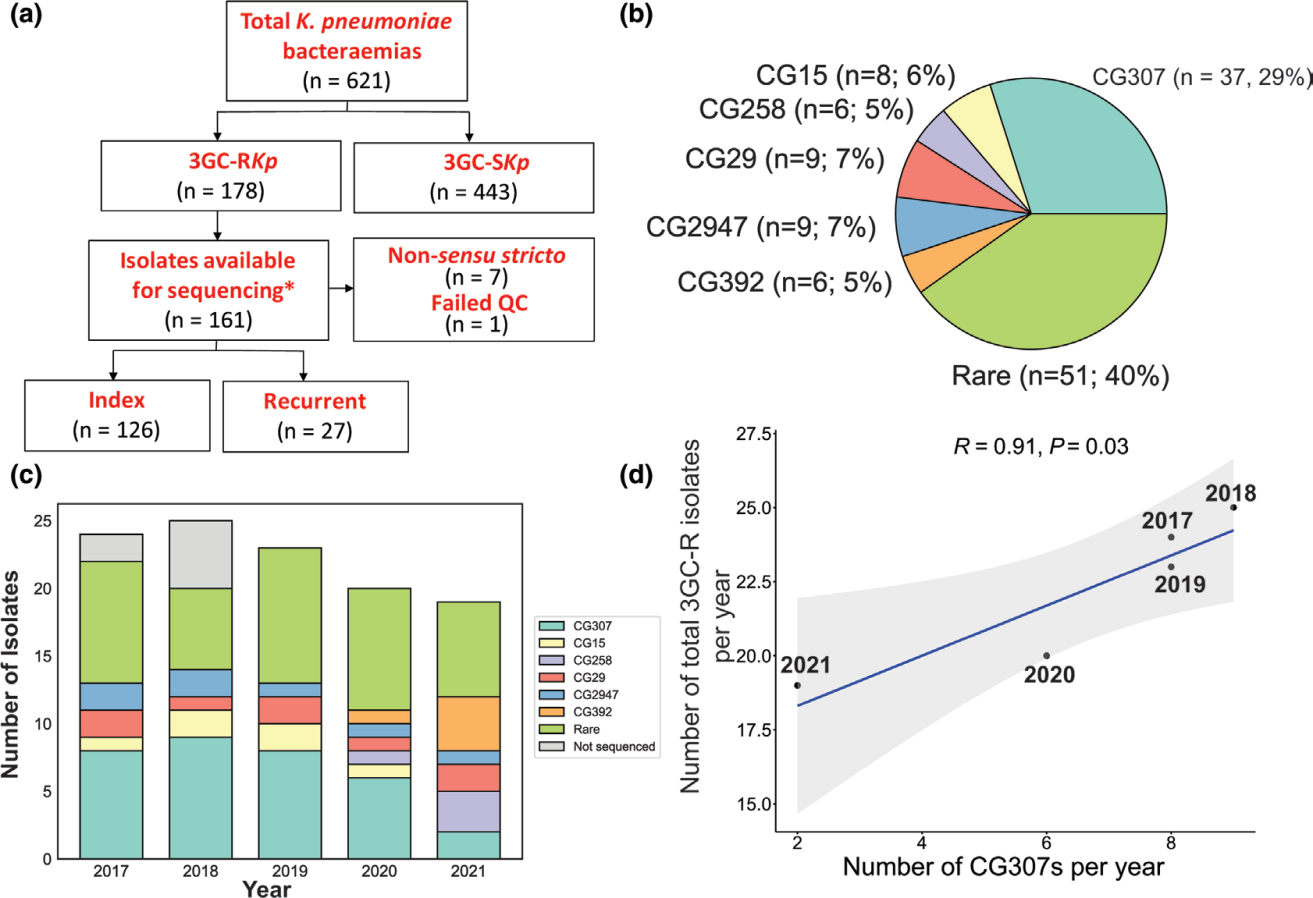
WGS workflow, CG distributions and trends of 3GC-R *K. pneumoniae* isolates. (**a**) Workflow of WGS inclusion/exclusion criteria for *K. pneumoniae* bacteraemia isolates. *, 27 isolates from a previous study (BioProject accession no. PRJNA836696) were included in our analyses. (**b**) Pie chart showing the CG distribution of index 3GC-R *K. pneumoniae* isolates. (**c**) CGs of index 3GC-R *K. pneumoniae* isolates stratified by year. The key indicates CGs by colour with grey representing index 3GC-R *K. pneumoniae* isolates that were not sequenced. (**d**) Positive correlation between sequenced CG307 isolates per year (*x*-axis) and the total number of index 3GC-R *K. pneumoniae* isolates from 2017 to 2021 (*y*-axis). Pearson’s correlation coefficient (*R*) with associated correlation test *P* value are reported in the figure.

Out of the 178 3GC-R *K. pneumoniae* causing bacteraemia during the study period, there were 90% (161/178) 3GC-R *K. pneumoniae* isolates available (Fig. 2a) that met our study’s inclusion criteria with 27 3GC-R *K. pneumoniae* isolates included from a previous study [21]. One index 3GC-R *K. pneumoniae* isolate had an assembly with genome size >7.5 Mbp and was excluded from the study. Furthermore, we identified seven index 3GC-R *K. pneumoniae* isolates (4 %) that belonged to *K. pneumoniae* species complex taxa that were not *K. pneumoniae sensu stricto* and, thus, were excluded from analysis [4]. For the remainder of the study, all references to *K. pneumoniae* refer to *K. pneumoniae sensu stricto* strains. Thus, we had a total of 153 isolates with WGS data available for analysis with 126 index and 27 recurrent 3GC-R *K. pneumoniae* isolates (Fig. 2a). Among the 126 index isolates, there was a total of 52 unique STs detected, highlighting the genomic diversity of our cohort. When grouping isolates by sharing at least five of seven loci from the Pasteur MLST scheme (i.e. CG designations), a total of 19 unique CGs were identified (Fig. 2b). The most predominant CG identified from our index 3GC-R *K. pneumoniae* isolates was CG307 (29 %; 37/126) with all 37 isolates sharing identical ST307 schema. Interestingly, we only detected 6 index 3GC-R *K. pneumoniae* isolates (5 %) that belonged to the pandemic CG258 lineage, with ST258 (*n*=2), ST11 (*n*=2) and ST395 (*n*=2) identified. Additional to CG258 and CG307 isolates, we only identified CGs with five or more index 3GC-R *K. pneumoniae* isolates for CG29 (9/126; 7 %), CG2947 (9/126; 7 %), CG15 (8/126; 6 %) and CG392 (6/126; 5 %), with the remaining 40 % (51/126) index 3GC-R *K. pneumoniae* isolates belonging to rarely identified CGs (i.e. <5 matching CG isolates detected). When stratified by full calendar years in our sampling frame (Fig. 2c), CG307 remained the most frequent CG detected per year, except in 2021 when CG392 (*n*=4) was the most common CG detected (NB 2016 and 2022 were excluded due to incomplete annual sampling). We observed a statistically significant positive correlation (Pearson correlation coefficient *r*=0.91; *P* value=0.03; Fig. 2d) between annual frequency of CG307 isolates collected and total 3GC-R *K. pneumoniae* infections detected per year, indicating that CG307 isolates may be the primary CG contributing to 3GC-R *K. pneumoniae* bacteraemia prevalence.

### High genomic diversity within index 3GC-R *K. pneumoniae* core-gene population structure

**Fig. 3.**
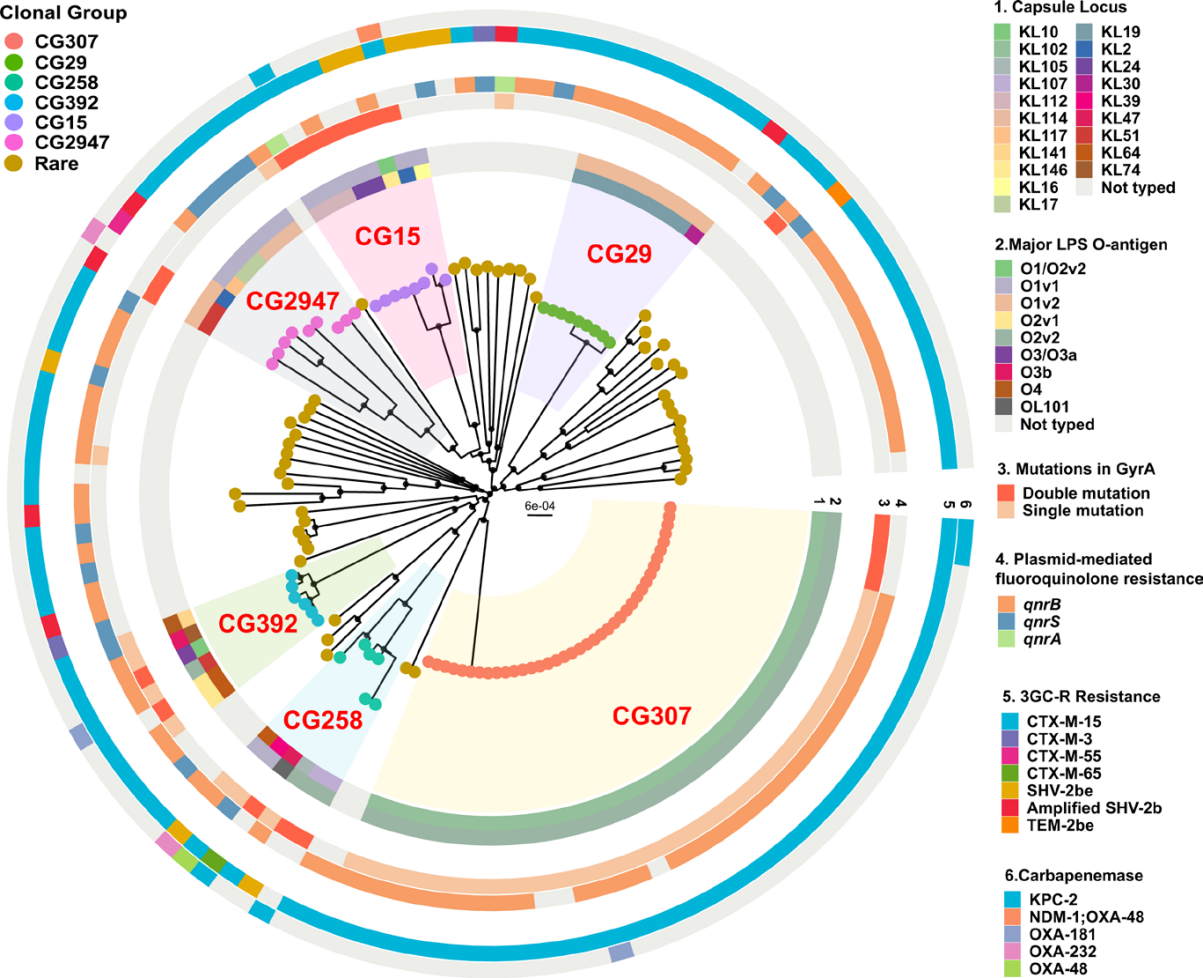
Core-gene maximum-likelihood inferred phylogenetic analysis of index 3GC-R *K. pneumoniae* strains. Phylogenetic tree is midpoint rooted with circular internal nodes representing 95 % UFBoot confidence values. Branch tips are labelled by CG with each CG highlighted within the tree structure. Rings are labelled as follows: (1) capsule type; (2) O-antigen; (3) GyrA polymorphisms; (4) plasmid-mediated FQR genes; (5) third-generation-cephalosporin-resistance genes; and (6) carbapenemase-encoding genes.

To gain further insights into 3GC-R *K. pneumoniae* population structure, we recreated a maximum-likelihood phylogeny inferred by a core-gene alignment of index 3GC-R *K. pneumoniae* isolates (*n*=126) and identified diverse, deep-branching lineages often observed in MDR *K. pneumoniae* [4]. Using a core-gene threshold definition of gene presence in ≥99 % of cohort, 16 % (3873/24 209) of the pangenome was ‘core’ gene content reflecting substantial pangenome diversity. The median pairwise nucleotide divergence was 0.58 % [median pairwise SNP distance (MPSD) = 21402] comparable to previous estimates [3, 20]. Although most CGs evidenced significant genetic diversity, there were clear clusters of closely related CG307 (MPSD=84) and CG29 (MPSD=15) isolates (Fig. 3). Both the CG307 (KL102) and CG29 (KL19) isolates had highly conserved capsule locus (KL) genes compared to the other CGs (Fig. 3). Similar to the KL locus, the O-locus was highly conserved in CG307s (O2v2), CG29 (O1v2) and CG15 (O1v1), when compared to the other CGs. When analysing primary determinants of virulence in *K. pneumoniae* [29, 44], the siderophore yersiniabactin locus (*ybt*) was present in 31 % of our 3GC-R *K. pneumoniae* population, consistent with published findings [44]. Less commonly detected virulence determinants were the genotoxin colibactin (*clb*, *n*=4), siderophore aerobactin (*iuc*, *n*=4), siderophore salmochelin (*iro*, *n*=1) and hypermucoidy *rmpADC* operon (*n*=1). One particularly interesting isolate belonged to ST412 (i.e. MB3190), which is a member of hypervirulent-associated CG23, which harboured the *rmpADC* operon in addition to *iro1* and *iuc1*, often carried on the FIB_k_ virulence plasmid KpVP-1 [44].

When focusing on the genetic determinants associated with 3GC-R *K. pneumoniae*, 94 % (119/126) harboured AMR genes encoding enzymes that are associated with a 3GC-R *K. pneumoniae* phenotype. The overwhelming majority of index 3GC-R *K. pneumoniae* isolates (*n*=109) harboured the ESBL-encoding gene *bla*_CTX-M_ with 96 % (105/109) harbouring the *bla*_CTX-M-15_ variant. We detected *bla*_SHV-2be_ variants in eight strains, only two of which were identified using the NCBI AMRFinderPlus database with our genomic assemblies; the remainder required the use of a short-read, *k*-mer mapping to database approach with KmerResistance to identify homologous *bla*_SHV-2b_ and *bla*_SHV-2be_ variants in the same strains. Additionally, convict identified five strains with amplification of *bla*_SHV-2b_ variants (i.e. gene copy number of ≥2.0×), with similar amplifications of penicillinase-encoding genes associated with a ‘false ESBL phenotype’ [45]. A carbapenemase-encoding gene was observed in 11 strains (9 %) (*bla*_KPC-2_ *n*=5; *bla*_OXA-48_ *n*=2; *bla*_OXA-181_ *n*=2; *bla*_OXA-232_ *n*=2) (Fig. 3). In total, a putative genetic mechanism for the third-generation-cephalosporin-resistance phenotype was present in 125/126 index 3GC-R *K. pneumoniae* isolates (99 %).

Given the extensive use of fluoroquinolones (FQs) as prophylaxis for neutropenic patients at our institution, in addition to associated FQ resistance (FQR) and ESBL-producing Gram-negative organisms [46], we next analysed FQ susceptibility and FQR mechanisms. Ciprofloxacin (CIP) non-susceptibility (CIP MIC ≥0.5 µg ml^−1^) was observed in 85 % (107/126) of index 3GC-R *K. pneumoniae* isolates. Quinolone-resistance-determining region (QRDR) mutations in *gyrA* and *parC* were observed in 51 % (64/126) and 41 % (52/126) of the strains, respectively, whereas the most commonly observed FQR mechanism in our cohort was the plasmid-mediated FQR gene *qnr*, primarily *qnrB1*, observed in 780% (101/126) of the strains. All CIP non-susceptible isolates had at least carriage of a *qnr* variant and/or one or more QRDR mutations. Single and double QRDR mutation for *gyrA* were almost exclusively observed in the major CGs such as CG307, CG258, CG392 and CG15, whereas rare CGs typically lacked *gyrA* QRDR mutations but did contain *qnr* genes (Fig. 3). The double mutation pattern *gyrA-83+87* was present in 14 strains, including four CG307s (Fig. 3).

### 3GC-R *K. pneumoniae* transmission and recurrence dynamics driven primarily by CG307 and CG29 clades

**Fig. 4.**
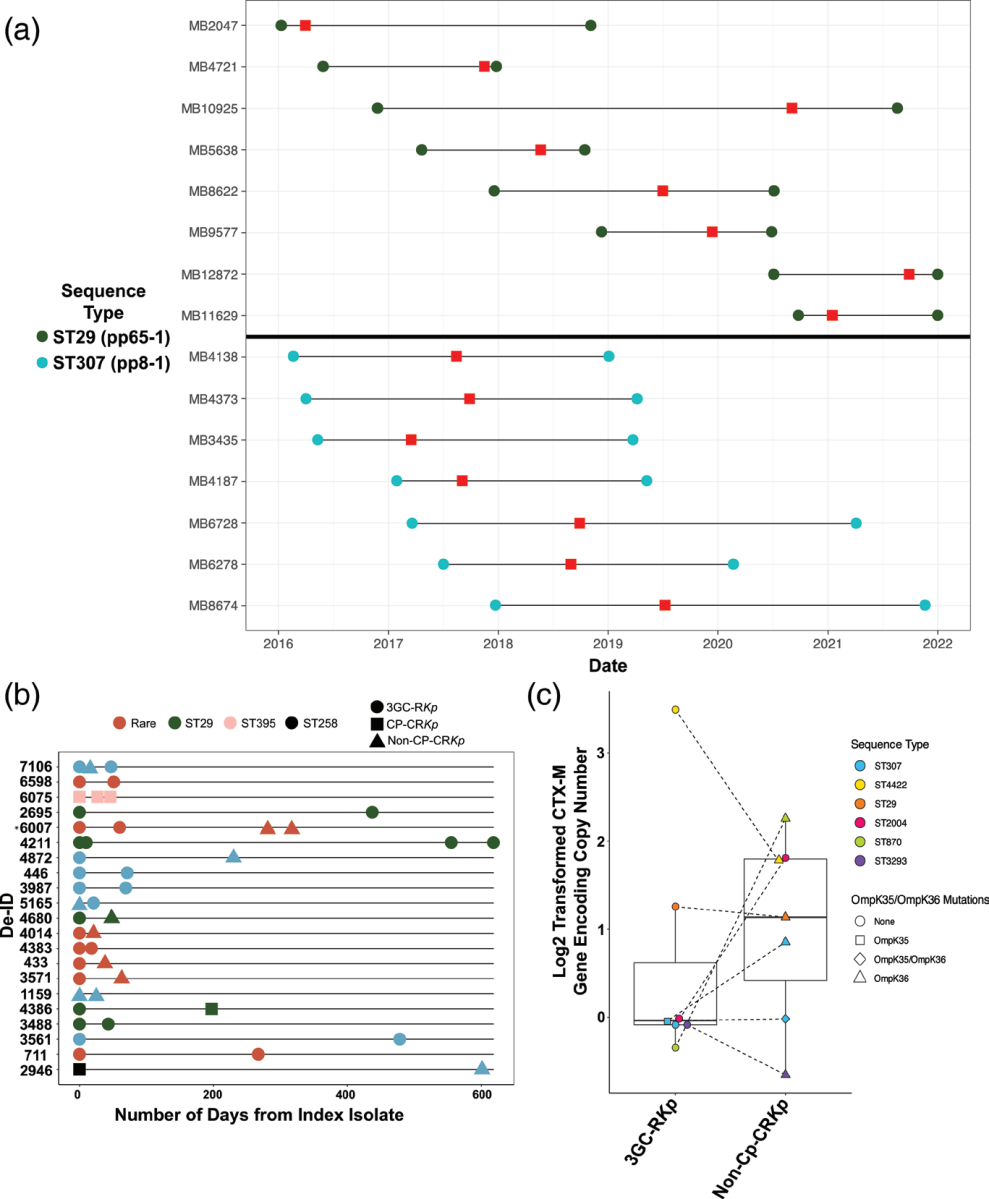
3GC-R *K. pneumoniae* transmission and recurrence dynamics. (**a**) Genetically related (i.e. <25 pairwise SNPs) isolates collected over time from unique patients for CG29 (top; dark green; transmission cluster pp8-1) and CG307 (bottom; light blue; transmission cluster pp65-1) clades. Circles represent the start and end of healthcare interactions at our institution for each respective patient, with the horizontal black line representing the duration of healthcare services. Red rectangles indicate collection date for each respective unique isolate. Note that patient–sample pair for MB12872 and MB11629 had end dates that went past 31/12/2021. (**b**) Index 3GC-R *K. pneumoniae* isolates with paired recurrent isolate (i.e. *K. pneumoniae* bacteraemia isolate collected >14 days from antecedent index isolate). Each row indicates a unique patient infection period. Duration of days from index to recurrent isolate is shown on the *x*-axis. Colour denotes ST and shape indicates phenotypes, as shown in the key. *, Note that patient 6007 had a ST conversion detailed in Table S5. 3GC-R*Kp*, third-generation-cephalosporin-resistant *K. pneumoniae*; CP-CR*Kp*, carbapenemase-producing carbapenem-resistant *K. pneumoniae*; non-CP-CR*Kp*, non-carbapenemase-producing carbapenem-resistant *K. pneumoniae.* (**c**) *bla*_CTX-M_ copy numbers and *ompK35/ompK36* mutation status in paired 3GC-R*Kp* (left) and recurrent non-CP-CR*Kp* (right) isolates. *y*-axis shows log2 transformed *bla*_CTX-M-15_ copy number estimates. Shapes and colours show *ompK35/ompK36* status and ST as delineated in the key.

Given the known capacity of 3GC-R *K. pneumoniae* to be transmitted in healthcare settings [2, 47], we used PopPUNK to identify nested populations of 3GC-R *K. pneumoniae* and subsequently performed PopPUNK cluster specific, core-genome-alignment inferred phylogenies masked for recombination to potentially identify transmission networks. We were able to identify 56 PopPUNK groups of which 14 included two or more isolates (i.e. 42 isolates were uniquely divergent from the full cohort, consistent with our CG population structure). From these 14 PopPUNK groups, we identified 28 3GC-R *K. pneumoniae* isolates from unique patients that differed by <25 pairwise SNPs (i.e. a SNP threshold that has been previously used to define potential *K. pneumoniae* transmission) [48] with a minimum of one other unique patient 3GC-R *K. pneumoniae* (Table S4). Potential strain transmission cluster sizes varied from two to eight isolates with a MPSD of 8 and range of 0–24 SNPs. Putative transmission clusters with genetically related pairwise isolates involved four distinct STs, namely ST307 (five clusters, 16 total isolates), ST29 (one cluster, 8 isolates), ST152 (one cluster, 2 isolates) and ST280 (one cluster, 2 isolates). Interestingly, 96 % (27/28) of index isolates from putative transmission clusters caused infections in patients with haematological malignancy compared to 66 % (65/98) non-clustered strains (χ^2^ *P* value <0.001). The two largest transmission clusters [ST307 (pp8-1), *n*=7; ST29 (pp65-1), *n*=8] both occurred over prolonged periods of time, 28 months for the ST307 pp8-1 cluster and nearly 60 months for the ST29 pp65-1 cluster in patients with multiple admissions and extensive contact with the healthcare system (Fig. 4a).

We sequenced 81 % (21/26) of the index 3GC-R *K. pneumoniae* bacteraemia isolates from patients who had one or more recurrent 3GC-R *K. pneumoniae* bacteraemias. We found that the first recurrent episode occurred on average 94 days following the initial 3GC-R *K. pneumoniae* bacteraemia episode (Fig. 4b, Table S5). The same ST as the index isolate caused 91 % of the recurrent infections. Consistent with carbapenems being the primary treatment for 3GC-R *K. pneumoniae*, eight isolates causing recurrent infections developed carbapenem resistance (Fig. 4b). The majority of strains (*n*=7) developed carbapenem resistance in the absence of a carbapenemase gene (i.e. were non-carbapenemase-producing carbapenem-resistant *K. pneumoniae* or non-CP-CR *K. pneumoniae*), whereas a single ST29 strain acquired *bla*_KPC-2_ (Fig. 4b). Through a combination of ONT long-read sequencing in addition to convict gene copy number estimates, we found that five out of the seven non-CP-CR *K. pneumoniae* recurrent isolates had evidence of *bla*_CTX-M_ gene amplification (i.e. *>*2× copies) (Fig. 4c). Additionally, mutations in *ompK*35 (*n*=1) and/or *ompK*36 (*n*=6) were observed in 6/7 (86 %) of the non-CP-CR *K. pneumoniae* isolates (Fig. 4c). These data indicate that recurrence of 3GC-R *K. pneumoniae* bacteraemia primarily occurs due to re-infection by the same strain, which has often developed carbapenem resistance through non-carbapenemase mechanisms.

### Identification of a novel lineage of CG307 causing bacteraemia at our institution

**Fig. 5.**
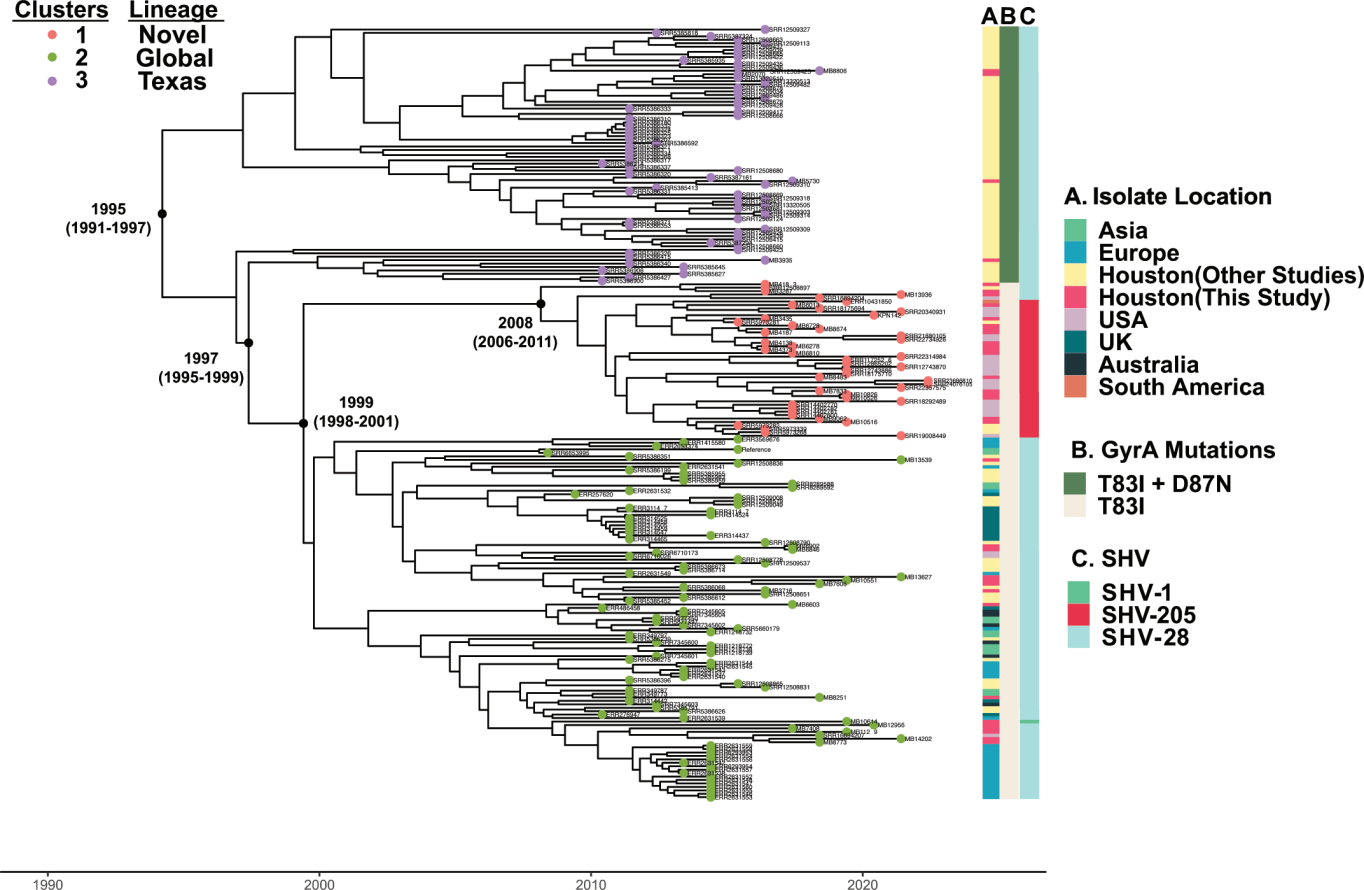
Identification of novel CG307 sub-clade (i.e. cluster 1) causing bacteraemia at our institution. Presented is a Bayesian dated, core-SNP, recombination masked, phylogeny of 224 CG307 strains using isolate Kp616 (BioSample accession no. SAMN08391417) as reference. Tip colours indicate hierBAPS predicted taxa with cluster 1 associated with a novel USA-based clade, cluster 2 associated with a global clade and cluster 3 associated with a Texas-specific clade. Years indicate predicted dates of divergence from internal nodes of interest as estimated using BactDating. Dates in parentheses represent the 95 %CI. Metadata presented in right-hand-side columns include: (A) geographical location of isolate collection, (B) presence of *gyrA* mutations and (C) chromosomal *bla*_SHV_ isoforms.

Given the stable detection of CG307 within the Houston region [7, 9, 20], including previously at our own institution [21], we further analysed CG307 isolates from our cohort (*n*=37) along with a worldwide distribution of geographically diverse CG307 isolates (*n*=187) publicly available from the NCBI. A Bayesian dated, recombination masked, phylogeny of 224 CG307s inferred from a core-genome-SNP alignment is presented in Fig. 5. Initial root-to-tip analysis of our CG307 cohort indicated a strong temporal signal (Fig. S1). Consistent with previous data, the CG307 splits into two primary clades, which have been previously described as Texas-specific (*n*=74) and ‘global’ clades (*n*=150). Within these respective CG307 clades, there were three nested sub-populations [10, 20]. The Texas-specific clade (cluster 3) harboured double GyrA-83I-87N mutations and 2× copies of *bla*_CTX-M-15_ that have previously been characterized (Fig. 5) [10]. Interestingly, only four CG307 isolates in our cohort clustered with the Texas-specific clade. In contrast, the majority (*n*=33) of our CG307 isolates belonged to the global cluster 2 (*n*=15) lineage described elsewhere [10] and a novel cluster 1 (*n*=18) lineage detected primarily in the USA (Fig. 5).

Our Bayesian dating analysis of our phylogeny estimates CG307 emerged in 1995 [95 % confidence interval (95 % CI): 1991–1997], consistent with a previous estimation of CG307 emergence in 1994 [10]. The date of divergence between the Texas-specific and CG307 clade that includes both global and novel lineages is estimated to have occurred in 1997 (95 %CI: 1995–2000) shortly after CG307 had emerged. Given that we observe CG307 from all three lineages in Houston, TX, suggests that CG307 may have originated from the Houston region, as hypothesized elsewhere [10]. When focusing on the global and novel clades, cluster 1 strains were estimated to have diverged from cluster 2 strains in approximately 1999 (95 %CI: 1998–2001), with cluster 1 isolates beginning to clonally expand near 2008 (95 %CI: 2006–2011) (Fig. 5). A conserved genomic feature of cluster 1 CG307 strains was the stable vertical transmission of *bla*_SHV-205_, a single amino acid variant of the chromosomal *bla*_SHV-1_ gene (Fig. 5). Consistent with our estimation of cluster 1 strains emerging near 2008, all publicly available cluster 1 strains with metadata were isolated from 2016 onwards with 15/25 isolated since 2020. All but one strain (Paraguay 2020) came from the USA, including such geographically diverse locales as Utah, Chicago and New York, and even included two strains isolated from dogs in Texas. Given that MDACC has a broad geographical patient distribution, we analysed the home location for each patient with a CG307 infection at our institution and found that regardless of the cluster designation of infecting isolates, most patients (31/37, 84 %) were from Texas and the majority were from the Houston area (54 %) (Fig. S2A). There were three patients from the USA who resided outside Texas, all of whom were infected by strains of cluster 1 (*n*=1) or cluster 2 (*n*=2) lineages (Fig. S2B). Three patients from outside the USA were infected with cluster 2 (*n*=3) lineages (Fig. S2B). These data indicate that the global (cluster 2) and novel, US-based (cluster 1) lineages of CG307 caused the majority of CG307 infections even among our patients from Houston and the rest of Texas.

### Differing accessory genome content across each of the CG307 clades

**Fig. 6.**
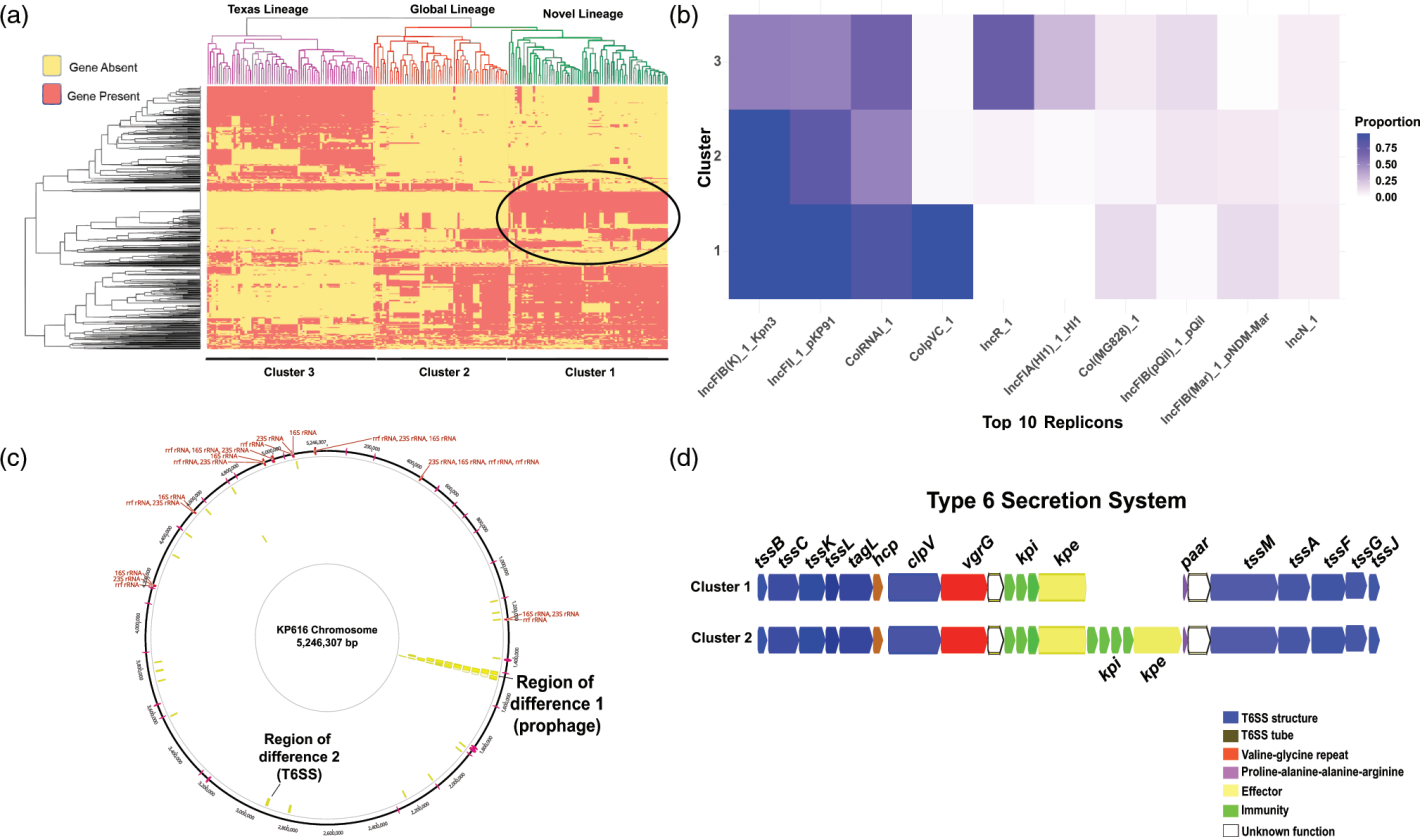
Assessment of accessory genome differences between CG307 strains. (**a**) Gene presence/absence heatmap representing the agglomerative hierarchical clustering of accessory genomes of CG307 strains. The circled region indicates genetic content unique to cluster 1 isolates. (**b**) Proportion heatmap of the most common replicon types identified using PlasmidFinder stratified by CG307 cluster. (**c**) Recombination hotspots (yellow blocks) of CG307 isolates using Kp616 as a reference and Gubbins predicted recombination sites output. Regions labelled on the chromosome indicate genomic context we identified that differed between cluster 1 and 2. (**d**) Region of difference 2 between CG307 cluster 1 and cluster 2 strains involving a T6SS. The labelling system for T6SS proteins is taken from another study [76] with a description of the colours used for putative gene functions shown in the key. Note that cluster 1 strains lack of a set of immunity/effector proteins present in cluster 2 strains.

To gain further insight into the various CG307 clusters, we used our Illumina data in conjunction with ONT long-read sequencing to dissect accessory genome content and determine genomic context of 3GC-R *K. pneumoniae* determinants. We subset all accessory genome content shared in greater than 5 % but less than 95 % of the CG307 population to look for gene presence/absence signals across our four clades and observed a demarcation across the four clusters (Fig. 6a). We noted that there were global and Texas-specific clade differences in replicon type detection, which has been described in previous literature (Fig. 6b) [20]. Cluster 1 and cluster 2 had an enrichment of IncFII (84 %) and IncFIB_K_ (97 %), whereas there were fewer cluster 3 isolates with either replicon detected [IncFII (54 %); IncFIB_K_ (52 %)] (Fig. 6b). These multireplicon F-type plasmid differences correlate with *bla*_CTX-M-15_ carriage differences between global and Texas-specific clades, as previously described [10, 20]. As shown in the supplementary data (Fig. S3A), both MB5730 and MB8806 (cluster 3) from the Texas-specific clade harboured two copies of *bla*_CTX-M-15_ inserted in the chromosome. Conversely, isolates MB7606 (cluster 2) and MB8674 (cluster 1) from the global clade harboured *bla*_CTX-M-15_ in association with IS*26* pseudo-compound transposons made of two or more IS*26* units with directly flanking IS*26* transposases on IncFIB_K_ plasmids (Fig. S3B).

We focused on accessory genome differences between the novel, US-based cluster 1 and global cluster 2 isolates to determine what was driving the potential emergence of cluster 1 in our region. We found that cluster 1 strains had a very high recombination to mutation (*r*/*m*) ratio of 16, suggesting that recombination events were driving diversity in this clade. We found two recombination blocks in cluster 1 isolates that appeared to be regions that differed with cluster 2 isolates (Fig. 6c). First, we found that, relative to cluster 2 strains, cluster 1 strains uniquely contain a 40 kbp prophage-like region that contains numerous putative regulatory proteins, as well as proteins of unknown function (Fig. 6c). Second, although both cluster 1 and cluster 2 strains contain a type VI secretion system (T6SS), cluster 1 strains lack a set of T6SS effector/immunity proteins that are present in cluster 2 strains (Fig. 6d) as well as in cluster 3 strains. Taken together, we conclude that the cluster 1 strains identified in our study are a distinct monophyletic group that has unique accessory gene content, as well as causing disease throughout the USA.

## Discussion

*K. pneumoniae* is a major contributor to healthcare-associated infections and can acquire resistance to a diverse array of antimicrobials, thereby rendering treatment problematic [1, 49, 50]. Given the limited molecular epidemiology of 3GC-R *K. pneumoniae* in the USA, we characterized 126 index and 27 recurrent 3GC-R *K. pneumoniae* bacteraemia isolates collected from 2016 to 2022 at our institution (Fig. 2a). Consistent with other worldwide MDR *K. pneumoniae* surveillance studies, we observed long-branching, genetically diverse 3GC-R *K. pneumoniae* isolates [22, 23, 48, 51–54]; however, there were clusters of ‘global problem clones’ including CG307, CG29 and CG15 isolates with evidence of limited transmission [4]. Moreover, through analysis of our patients and publicly available genomes, we have identified a previously uncharacterized *K. pneumoniae* CG307 sub-clade (i.e. cluster 1) that caused 14 % of our index 3GC-R *K. pneumoniae* infections and has been isolated recently from multiple locations across the USA [26, 54].

A major impetus for our study was reports of a recent expansion of 3GC-R *K. pneumoniae* in the USA [12, 55]; however, prospective data from active 3GC-R *K. pneumoniae* surveillance is limited and, thus, increases in incident 3GC-R *K. pneumoniae* infections remain unclear [56]. During our study time frame, we observed fairly stable absolute frequencies, as well as admission-adjusted prevalence of 3GC-R *K. pneumoniae* bacteraemias (Fig. 1). It has been suggested that increases in 3GC-R *Enterobacterales* infections in the USA have been largely driven by community-onset cases [49]. The genetic diversity of 3GC-R *K. pneumoniae* isolates (Fig. 3), in conjunction with seasonal prevalence differences of 3GC-R *K. pneumoniae* infections (Fig. 1b, c), suggest a possible community-wide dissemination in our region. These data are similar to those recently described for a hospital network in Australia, in which comprehensive WGS of *K. pneumoniae* found that the majority of infections were caused by widespread circulation in the community rather than hospital transmission [48]. Consistent with our observation, 3GC-R *K. pneumoniae* infections tended to occur in the warmer second half of the calendar year compared to the first half of the year, a multi-site surveillance study across several continents found that environmental factors associated with warmer months may increase incidence rates of *K. pneumoniae* infections [57]. Nevertheless, similar to a recent analysis on 3GC-R *E. coli* prevalence at our institution [43], we observed a statistically significant association only for 3GC-R *K. pneumoniae* but not 3GC-S *K. pneumoniae* infections. Thus, our results taken together with previous studies suggest that limiting 3GC-R *K. pneumoniae* acquisition outside of the hospital is likely to be critical to mitigation efforts.

Although we had a long-branching, genetically diverse cohort of 3GC-R *K. pneumoniae* isolates (Figs 2 and 3), we also observed numerous instances of potential hospital-based 3GC-R *K. pneumoniae* transmission (Fig. 4a). It is worth noting that given we only sampled 3GC-R *K. pneumoniae* isolates collected from bacteraemia infections, we are likely underestimating the true scope of transmission that could be occurring at our institution. Interestingly, transmission clusters almost exclusively were due to the ‘problem clones’ CG307 and CG29 (Fig. 4a), and involved patients with haematological malignancy, likely because of the propensity of such individuals to develop bacteraemias following initial colonization [58]. Outbreaks of these two global problem clones have been described [59], although whether such transmission is happening due to direct human contact or being acquired from the hospital environment is not currently known. Interestingly, for both CG307 and CG29 strains, we observed highly genetically related bacteria causing infections over a several years’ time frame (Fig. 4a), suggesting potential environmental sources as has been observed for *Pseudomonas* spp. [60] and vancomycin-resistant *Enterococcus faecium* [61]. The lack of clear epidemiological links between infected patients means that standard infection control approaches would be unlikely to detect such transmission events, emphasizing the potential complementary role that WGS could play in infection control efforts if real-time data were available [62].

Recurrence is a major concern following treatment of 3GC-R *K. pneumoniae* infections [63, 64], and indeed we observed an approximate 20 % recurrence rate when just considering bacteraemias. The recurrent strain was nearly always the same ST and highly genetically related to the index isolate. suggesting re-infection rather than acquisition of a new 3GC-R *K. pneumoniae* strain. Interestingly, the recurrent organism could either retain a 3GC-R, carbapenem-susceptible phenotype, or transition to carbapenem resistance, usually without acquisition of a carbapenemase (Fig. 4b). Although data are limited, a few studies have found that intravenous carbapenem administration does not reliably eradicate ESBL-positive *Enterobacterales* from the intestinal tract, presumably due to low carbapenem stool concentrations [65, 66]. Alternatively, it has been recently demonstrated that *Enterobacter* spp. can enter into a cell-well-deficient spheroplast form capable of surviving carbapenem exposure and return to normal growth once carbapenem pressure is removed; thus, carbapenem ‘tolerance’ could have accounted for strain persistence [67]. 3GC-R *K. pneumoniae* isolates can also develop outright resistance to carbapenems in the absence of carbapenemase by limiting drug influx through porin mutations along with hyperproduction of *bla*_CTX-M_-encoding enzymes, which we were able to demonstrate in multiple serial isolates using a long-read sequencing approach [21]. Remarkably, we collected many recurrent isolates months or years from the index isolate collection date (Fig. 4b) with minimal pairwise SNP differences (i.e. <10 SNPs), suggesting the long duration of colonization achievable by 3GC-R *K. pneumoniae* [68]. We envision that a strategy of targeted *K. pneumoniae* gastrointestinal tract decolonization, such as using CRISPR-based technologies, could be part of the future treatment of 3GC-R *K. pneumoniae* infections [69].

We were intrigued by the paucity of Texas-specific CG307 isolates in contrast to the global and novel, US-based CG307 clades collected in our cohort, in addition to the relatively low number of CG258 isolates detected (Fig. 2). A study from a Houston-wide hospital system analysing ESBL *K. pneumoniae* strains collected from 2011 to 2015 found similar numbers of CG307 and CG258 strains, an unexpected result given the predominant prevalence of CG258 in most USA investigations [5, 9, 19]. A comparative genomics analysis of CG307 isolates found that the Texas-specific clade was genetically distinct from other CG307 strains circulating worldwide, with multiple chromosomal insertions of *bla*_CTX-M-15_ and a fixed double GyrA mutation as a predominant genomic feature of the Texas-specific CG307 clade [10]. Another Houston-based study found divergent accessory genomes between CG258 and CG307 with greater plasmid content in the latter, suggesting that CG307 isolates had a potential greater capacity to adapt to selective pressures [20]. Nevertheless, this study found very few of the global CG307 clade circulating in their particular hospital system [20]. The reason for our finding that only a small fraction of CG307 isolates belong to the Texas lineage is not clear, but might be due to temporal variation given our most recent sampling time frame. Support for this hypothesis comes from the relatively recent isolation of CG307 strains from across multiple USA locales, including Central Texas [26] and Mississippi, that cluster together with the majority of our novel, USA-based CG307 isolates. These isolates have a distinct chromosomal SHV, lack the multiple copies of chromosomal *bla*_CTX-M-15_ hypothesized to be important to the success of the Texas-specific lineage, and contain a unique accessory genome relative to other CG307 strains (Figs 5 and 6). Our findings mirror similar, recent clonal expansions of CG307 strains from the global lineage observed in Wales and Norway, suggesting that the USA may be in the early stages of dissemination of this USA-based CG307 sub-clade [22, 23].

We identified two major events distinguishing the global CG307 cluster 1 and cluster 2 isolates. The cluster 1 strains contained a prophage that did not display any clear virulence factors, although numerous ORFs encoded proteins of unknown function and acquisition of exogenous DNA has been shown to influence the transcriptome of distant genes, possibly through the presence of transcriptional regulators in the acquired DNA [70, 71]. Additionally, relative to cluster 1, cluster 2 strains had a distinct set of effector/immunity proteins from a T6SS (Fig. 6d). T6SSs function as injection machinery that can directly puncture both eukaryotic and prokaryotic membranes, and have been identified as contributing to *K. pneumoniae* pulmonary infection, gastrointestinal colonization, bacterial competition and liver abscess formation [72–75]. Future work will be necessary to further functionally characterize potential differences in virulence and resistance factors contributing to each CG307 clades’ respective success.

Although our study had many strengths, there are some limitations. First, our isolates were recovered from a single centre and, thus, the generalizability of our findings is not known; however, our cancer centre draws from a large geographical region, and Houston has been a notable centre of 3GC-R *K. pneumoniae* infections [9, 20]. Second, we only analysed bloodstream infections, which limits our results’ generalizability when it comes to other 3GC-R *K. pneumoniae* infectious sources; nevertheless, given the invasive nature of bacteraemia, this reduces likelihood of identifying false-positive infections. Finally, the genetic diversity of 3GC-R *K. pneumoniae* isolates meant that we could only analyse in-depth a few CGs even with a sufficiently large sample size of over 150 3GC-R *K. pneumoniae* samples sequenced.

In summary, we present a contemporary, comparative genomics-based analysis of 3GC-R *K. pneumoniae* causing invasive disease in immunocompromised patients. We found that a previously unrecognized, USA-based CG307 sub-clade is causing significant disease both in Houston and in diverse locations across the USA, suggesting ongoing spread of an MDR *K. pneumoniae* strain with unique accessory genome content. Our findings emphasize the continued need for 3GC-R *Enterobacterales* surveillance in addition to the efforts made to track carbapenem-resistant strains.

## Supplementary Data

Supplementary material 1

Supplementary material 2

## References

[1] Murray CJL, Ikuta KS, Sharara F, Swetschinski L, Robles Aguilar G (2022). Global burden of bacterial antimicrobial resistance in 2019: a systematic analysis. The Lancet.

[2] Mohd Asri NA, Ahmad S, Mohamud R, Mohd Hanafi N, Mohd Zaidi NF (2021). Global prevalence of nosocomial multidrug-resistant *Klebsiella pneumoniae*: a systematic review and meta-analysis. Antibiotics.

[3] Holt KE, Wertheim H, Zadoks RN, Baker S, Whitehouse CA (2015). Genomic analysis of diversity, population structure, virulence, and antimicrobial resistance in *Klebsiella pneumoniae*, an urgent threat to public health. Proc Natl Acad Sci U S A.

[4] Wyres KL, Lam MMC, Holt KE (2020). Population genomics of *Klebsiella pneumoniae*. Nat Rev Microbiol.

[5] Kitchel B, Rasheed JK, Patel JB, Srinivasan A, Navon-Venezia S (2009). Molecular epidemiology of KPC-producing *Klebsiella pneumoniae* isolates in the United States: clonal expansion of multilocus sequence type 258. Antimicrob Agents Chemother.

[6] Bowers JR, Kitchel B, Driebe EM, MacCannell DR, Roe C (2015). Genomic analysis of the emergence and rapid global dissemination of the clonal group 258 *Klebsiella pneumoniae* pandemic. PLoS One.

[7] Castanheira M, Farrell SE, Wanger A, Rolston KV, Jones RN (2013). Rapid expansion of KPC-2-producing *Klebsiella pneumoniae* isolates in two Texas hospitals due to clonal spread of ST258 and ST307 lineages. Microb Drug Resist.

[8] Villa L, Feudi C, Fortini D, Brisse S, Passet V (2017). Diversity, virulence, and antimicrobial resistance of the KPC-producing *Klebsiella pneumoniae* ST307 clone. Microb Genom.

[9] Long SW, Olsen RJ, Eagar TN, Beres SB, Zhao P (2017). Population genomic analysis of 1,777 extended-spectrum beta-lactamase-producing *Klebsiella pneumoniae* isolates. MBio.

[10] Wyres KL, Hawkey J, Hetland MAK, Fostervold A, Wick RR (2019). Emergence and rapid global dissemination of CTX-M-15-associated *Klebsiella pneumoniae* strain ST307. J Antimicrob Chemother.

[11] Queenan AM, Bush K (2007). Carbapenemases: the versatile beta-lactamases. Clin Microbiol Rev.

[12] Castanheira M, Kimbrough JH, DeVries S, Mendes RE, Sader HS (2023). Trends of β-lactamase occurrence among *Escherichia coli* and *Klebsiella pneumoniae* in United States hospitals during a 5-year period and activity of antimicrobial agents against isolates stratified by β-lactamase type. Open Forum Infect Dis.

[13] Rojas LJ, Weinstock GM, De La Cadena E, Diaz L, Rios R (2017). An analysis of the epidemic of *Klebsiella pneumoniae* carbapenemase-producing *K. pneumoniae*: convergence of two evolutionary mechanisms creates the “perfect storm.”. J Infect Dis.

[14] Wang M, Earley M, Chen L, Hanson BM, Yu Y (2022). Clinical outcomes and bacterial characteristics of carbapenem-resistant *Klebsiella pneumoniae* complex among patients from different global regions (CRACKLE-2): a prospective, multicentre, cohort study. Lancet Infect Dis.

[15] David S, Reuter S, Harris SR, Glasner C, Feltwell T (2019). Epidemic of carbapenem-resistant *Klebsiella pneumoniae* in Europe is driven by nosocomial spread. Nat Microbiol.

[16] Cerqueira GC, Earl AM, Ernst CM, Grad YH, Dekker JP (2017). Multi-institute analysis of carbapenem resistance reveals remarkable diversity, unexplained mechanisms, and limited clonal outbreaks. Proc Natl Acad Sci U S A.

[17] Satlin MJ, Chen L, Patel G, Gomez-Simmonds A, Weston G (2017). Multicenter clinical and molecular epidemiological analysis of bacteremia due to carbapenem-resistant Enterobacteriaceae (CRE) in the CRE epicenter of the United States. Antimicrob Agents Chemother.

[18] van Duin D, Arias CA, Komarow L, Chen L, Hanson BM (2020). Molecular and clinical epidemiology of carbapenem-resistant Enterobacterales in the USA (CRACKLE-2): a prospective cohort study. Lancet Infect Dis.

[19] Deleo FR, Chen L, Porcella SF, Martens CA, Kobayashi SD (2014). Molecular dissection of the evolution of carbapenem-resistant multilocus sequence type 258 *Klebsiella pneumoniae*. Proc Natl Acad Sci U S A.

[20] Shropshire WC, Dinh AQ, Earley M, Komarow L, Panesso D (2022). Accessory genomes drive independent spread of carbapenem-resistant *Klebsiella pneumoniae* clonal groups 258 and 307 in Houston, TX. mBio.

[21] Shropshire WC, Konovalova A, McDaneld P, Gohel M, Strope B (2022). Systematic analysis of mobile genetic elements mediating β-lactamase gene amplification in noncarbapenemase-producing carbapenem-resistant *Enterobacterales* bloodstream infections. mSystems.

[22] Fostervold A, Hetland MAK, Bakksjø R, Bernhoff E, Holt KE (2022). A nationwide genomic study of clinical *Klebsiella pneumoniae* in Norway 2001-15: introduction and spread of ESBLs facilitated by clonal groups CG15 and CG307. J Antimicrob Chemother.

[23] David S, Mentasti M, Sands K, Portal E, Graham L (2023). Genomic surveillance of multidrug-resistant *Klebsiella* in Wales reveals persistent spread of *Klebsiella pneumoniae* ST307 and adaptive evolution of pOXA-48-like plasmids. Microb Genom.

[24] Heiden SE, Hübner N-O, Bohnert JA, Heidecke C-D, Kramer A (2020). A *Klebsiella pneumoniae* ST307 outbreak clone from Germany demonstrates features of extensive drug resistance, hypermucoviscosity, and enhanced iron acquisition. Genome Med.

[25] Black CA, So W, Dallas SS, Gawrys G, Benavides R (2020). Predominance of non-carbapenemase producing carbapenem-resistant Enterobacterales in South Texas. Front Microbiol.

[26] Parker JK, Gu R, Estrera GA, Kirkpatrick B, Rose DT (2023). Carbapenem-resistant and ESBL-producing enterobacterales emerging in Central Texas. Infect Drug Resist.

[27] CLSI (2023). Performance Standards for Antimicrobial Susceptibility Testing, 33rd edn.

[28] Prjibelski A, Antipov D, Meleshko D, Lapidus A, Korobeynikov A (2020). Using SPAdes *de novo* assembler. Curr Protoc Bioinformatics.

[29] Lam MMC, Wick RR, Watts SC, Cerdeira LT, Wyres KL (2021). A genomic surveillance framework and genotyping tool for *Klebsiella pneumoniae* and its related species complex. Nat Commun.

[30] Wyres KL, Wick RR, Gorrie C, Jenney A, Follador R (2016). Identification of *Klebsiella* capsule synthesis loci from whole genome data. Microb Genom.

[31] Hennart M, Guglielmini J, Bridel S, Maiden MCJ, Jolley KA (2022). A dual barcoding approach to bacterial strain nomenclature: genomic taxonomy of *Klebsiella pneumoniae* strains. Mol Biol Evol.

[32] Clausen P, Zankari E, Aarestrup FM, Lund O (2016). Benchmarking of methods for identification of antimicrobial resistance genes in bacterial whole genome data. J Antimicrob Chemother.

[33] Clausen P, Aarestrup FM, Lund O (2018). Rapid and precise alignment of raw reads against redundant databases with KMA. BMC Bioinformatics.

[34] Robinson JT, Thorvaldsdóttir H, Winckler W, Guttman M, Lander ES (2011). Integrative genomics viewer. Nat Biotechnol.

[35] Seemann T (2014). Prokka: rapid prokaryotic genome annotation. Bioinformatics.

[36] Page AJ, Cummins CA, Hunt M, Wong VK, Reuter S (2015). Roary: rapid large-scale prokaryote pan genome analysis. Bioinformatics.

[37] Katoh K, Standley DM (2013). MAFFT multiple sequence alignment software version 7: improvements in performance and usability. Mol Biol Evol.

[38] Minh BQ, Schmidt HA, Chernomor O, Schrempf D, Woodhams MD (2020). IQ-TREE 2: new models and efficient methods for phylogenetic inference in the genomic era. Mol Biol Evol.

[39] Hoang DT, Chernomor O, von Haeseler A, Minh BQ, Vinh LS (2018). UFBoot2: improving the ultrafast bootstrap approximation. Mol Biol Evol.

[40] Lees JA, Harris SR, Tonkin-Hill G, Gladstone RA, Lo SW (2019). Fast and flexible bacterial genomic epidemiology with PopPUNK. Genome Res.

[41] Tonkin-Hill G, Lees JA, Bentley SD, Frost SDW, Corander J (2018). RhierBAPS: an R implementation of the population clustering algorithm hierBAPS. Wellcome Open Res.

[42] Grant JR, Enns E, Marinier E, Mandal A, Herman EK (2023). Proksee: in-depth characterization and visualization of bacterial genomes. Nucleic Acids Res.

[43] Shropshire WC, Strope B, Selvaraj Anand S, Bremer J, McDaneld P (2023). Temporal dynamics of genetically heterogeneous extended-spectrum cephalosporin-resistant *Escherichia coli* bloodstream infections. mSphere.

[44] Lam MMC, Wick RR, Wyres KL, Gorrie CL, Judd LM (2018). Genetic diversity, mobilisation and spread of the yersiniabactin-encoding mobile element ICEKp in *Klebsiella pneumoniae* populations. Microb Genom.

[45] Beceiro A, Maharjan S, Gaulton T, Doumith M, Soares NC (2011). False extended-spectrum β-lactamase phenotype in clinical isolates of *Escherichia coli* associated with increased expression of OXA-1 or TEM-1 penicillinases and loss of porins. J Antimicrob Chemother.

[46] Wiener ES, Heil EL, Hynicka LM, Johnson JK (2016). Are fluoroquinolones appropriate for the treatment of extended-spectrum β-lactamase-producing Gram-negative bacilli?. J Pharm Technol.

[47] Weber A, Neffe L, Diaz LAP, Thoma N, Aghdassi SJS (2023). Analysis of transmission-related third-generation cephalosporin-resistant Enterobacterales by electronic data mining and core genome multi-locus sequence typing. J Hosp Infect.

[48] Gorrie CL, Mirčeta M, Wick RR, Judd LM, Lam MMC (2022). Genomic dissection of *Klebsiella pneumoniae* infections in hospital patients reveals insights into an opportunistic pathogen. Nat Commun.

[49] Jernigan JA, Hatfield KM, Wolford H, Nelson RE, Olubajo B (2020). Multidrug-resistant bacterial infections in U.S. hospitalized patients, 2012–2017. N Engl J Med.

[50] Arcari G, Carattoli A (2023). Global spread and evolutionary convergence of multidrug-resistant and hypervirulent *Klebsiella pneumoniae* high-risk clones. Pathog Glob Health.

[51] Ljungquist O, Haldorsen B, Pöntinen AK, Janice J, Josefsen EH (2023). Nationwide, population-based observational study of the molecular epidemiology and temporal trend of carbapenemase-producing Enterobacterales in Norway, 2015 to 2021. Euro Surveill.

[52] Garcia-Gonzalez N, Fuster B, Tormo N, Salvador C, Gimeno C (2023). Genomic analysis of the initial dissemination of carbapenem-resistant *Klebsiella pneumoniae* clones in a tertiary hospital. Microb Genom.

[53] Thorpe HA, Booton R, Kallonen T, Gibbon MJ, Couto N (2022). A large-scale genomic snapshot of *Klebsiella* spp. isolates in Northern Italy reveals limited transmission between clinical and non-clinical settings. Nat Microbiol.

[54] Kochan TJ, Nozick SH, Medernach RL, Cheung BH, Gatesy SWM (2022). Genomic surveillance for multidrug-resistant or hypervirulent *Klebsiella pneumoniae* among United States bloodstream isolates. BMC Infect Dis.

[55] McDanel J, Schweizer M, Crabb V, Nelson R, Samore M (2017). Incidence of extended-spectrum β-lactamase (ESBL)-producing *Escherichia coli* and *Klebsiella* infections in the United States: a systematic literature review. Infect Control Hosp Epidemiol.

[56] Duffy N, Karlsson M, Reses HE, Campbell D, Daniels J (2022). Epidemiology of extended-spectrum β-lactamase-producing Enterobacterales in five US sites participating in the Emerging Infections Program, 2017. Infect Control Hosp Epidemiol.

[57] Anderson DJ, Richet H, Chen LF, Spelman DW, Hung Y-J (2008). Seasonal variation in *Klebsiella pneumoniae* bloodstream infection on 4 continents. J Infect Dis.

[58] de la Court JR, Woudt SHS, Schoffelen AF, Heijmans J, de Jonge NA (2022). Third-generation cephalosporin resistant gram-negative bacteraemia in patients with haematological malignancy; an 11-year multi-centre retrospective study. Ann Clin Microbiol Antimicrob.

[59] Gorrie CL, Mirceta M, Wick RR, Judd LM, Wyres KL (2018). Antimicrobial-resistant *Klebsiella pneumoniae* carriage and infection in specialized geriatric care wards linked to acquisition in the referring hospital. Clin Infect Dis.

[60] Diorio-Toth L, Wallace MA, Farnsworth CW, Wang B, Gul D (2023). Intensive care unit sinks are persistently colonized with multidrug resistant bacteria and mobilizable, resistance-conferring plasmids. mSystems.

[61] El Haddad L, Hanson BM, Arias CA, Ghantoji SS, Harb CP (2021). Emergence and transmission of daptomycin and vancomycin-resistant enterococci between patients and hospital rooms. Clin Infect Dis.

[62] Sundermann AJ, Chen J, Kumar P, Ayres AM, Cho ST (2022). Whole-genome sequencing surveillance and machine learning of the electronic health record for enhanced healthcare outbreak detection. Clin Infect Dis.

[63] Mert D, Iskender G, Kolgelier S, Ertek M (2023). Evaluation of risk factors, causative pathogens, and treatment in recurrent percutaneous nephrostomy catheter-related urinary tract infections in cancer patients. Medicine.

[64] Refay SM, Ahmed EH, Abd ELzaher AR, Morsy AM, Yasser MM (2022). Risk of drug resistance and repeated infection with *Klebsiella pneumoniae* and *Escherichia coli* in intensive care unit cancer patients. Comb Chem High Throughput Screen.

[65] Djukovic A, González-Barberá EM, Sanz J, Artacho A, Peñaranda I (2020). High heterogeneity of multidrug-resistant *Enterobacteriaceae* fecal levels in hospitalized patients is partially driven by intravenous β-lactams. Antimicrob Agents Chemother.

[66] Gundes S, Arisoy AE, Kolayli F, Karaali E, Turker G (2005). An outbreak of SHV-5 producing *Klebsiella pneumoniae* in a neonatal intensive care unit; meropenem failed to avoid fecal colonization. New Microbiol.

[67] Murtha AN, Kazi MI, Schargel RD, Cross T, Fihn C (2022). High-level carbapenem tolerance requires antibiotic-induced outer membrane modifications. PLoS Pathog.

[68] Herrera S, Torralbo B, Herranz S, Bernal-Maurandi J, Rubio E (2023). Carriage of multidrug-resistant Gram-negative bacilli: duration and risk factors. Eur J Clin Microbiol Infect Dis.

[69] Campos-Madueno EI, Moradi M, Eddoubaji Y, Shahi F, Moradi S (2023). Intestinal colonization with multidrug-resistant Enterobacterales: screening, epidemiology, clinical impact, and strategies to decolonize carriers. Eur J Clin Microbiol Infect Dis.

[70] Roshika R, Jain I, Medicielo J, Wächter J, Danger JL (2021). The RD2 pathogenicity Island modifies the disease potential of the group A *Streptococcus*. Infect Immun.

[71] Vega LA, Sanson MA, Cubria MB, Regmi S, Shah BJ (2022). The integrative conjugative element ICESpyM92 contributes to pathogenicity of emergent antimicrobial-resistant *emm92* group A *Streptococcus*. Infect Immun.

[72] Wong Fok Lung T, Charytonowicz D, Beaumont KG, Shah SS, Sridhar SH (2022). *Klebsiella pneumoniae* induces host metabolic stress that promotes tolerance to pulmonary infection. Cell Metab.

[73] Merciecca T, Bornes S, Nakusi L, Theil S, Rendueles O (2022). Role of *Klebsiella pneumoniae* type VI secretion system (T6SS) in long-term gastrointestinal colonization. Sci Rep.

[74] Storey D, McNally A, Åstrand M, Sa-Pessoa Graca Santos J, Rodriguez-Escudero I (2020). *Klebsiella pneumoniae* type VI secretion system-mediated microbial competition is PhoPQ controlled and reactive oxygen species dependent. PLoS Pathog.

[75] Wang H, Guo Y, Liu Z, Chang Z (2023). The type VI secretion system contributes to the invasiveness of liver abscess caused by *Klebsiella pneumoniae*. J Infect Dis.

[76] Li W, Liu X, Tsui W, Xu A, Li D (2022). Identification and comparative genomic analysis of type VI secretion systems and effectors in *Klebsiella pneumoniae*. Front Microbiol.

