# Supplementary material for: Identification of a novel CG307 sub-clade in third-generation-cephalosporin-resistant Klebsiella pneumoniae causing invasive infections in the USA
Source: Microb Genom. 2024 Feb 26;10(2):001201. doi: 10.1099/mgen.0.001201 (PMC10926705; doi:10.1099/mgen.0.001201)
Supplement: Supplementary material 1 [file mgen-10-1201-s001.pdf]

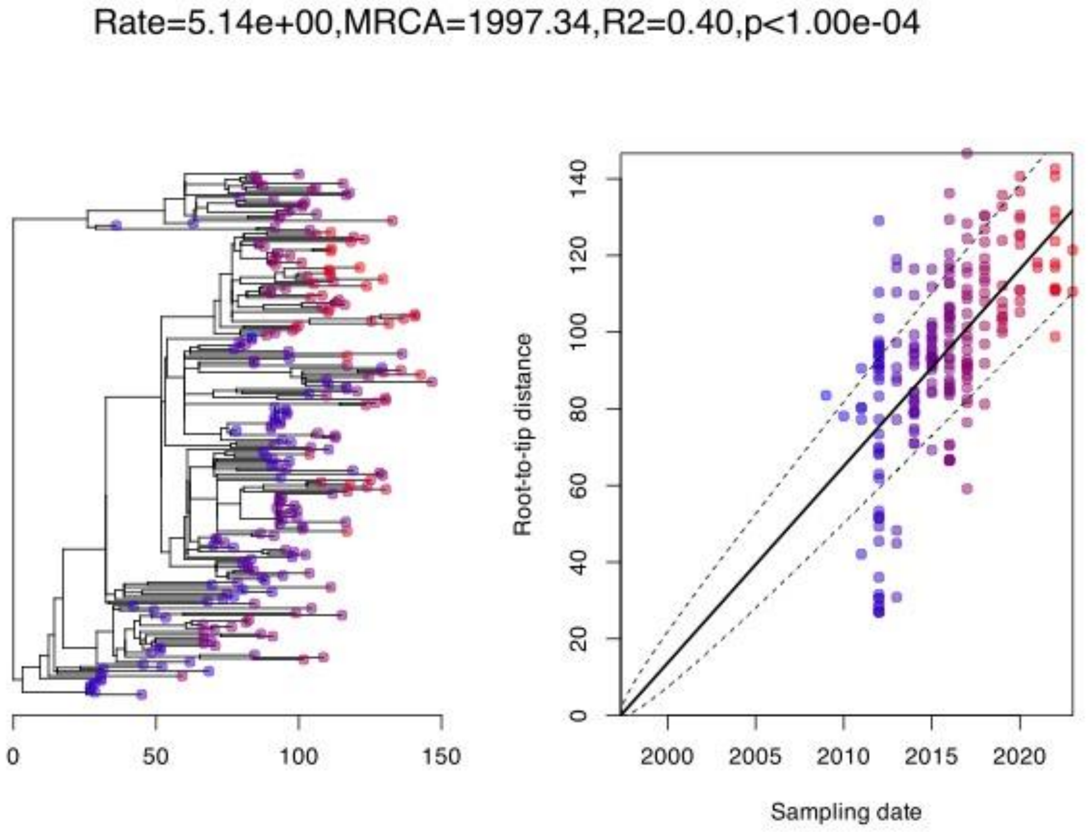

Fig. S1. Initial root-to-tip correlation analysis of 224 CG307 isolates. Initial test of Gubbins phylogeny with an estimate mu=5.14 substitutions/genome, an MRCA = 1997, and significant test of temporal signal. Model testing with a null temporal signal (*i.e.,* all sampling dates equal) phylogeny indicates temporal signal model (model=carc) was significant. We found a strong correlation between the sampling dates and the root-to-tip distances in addition to the significant temporal signal (*P*-value<0.05). We selected for 10000 Markov Chain Monte Carlo simulations using a 'carc' model to obtain the dated phylogeny.

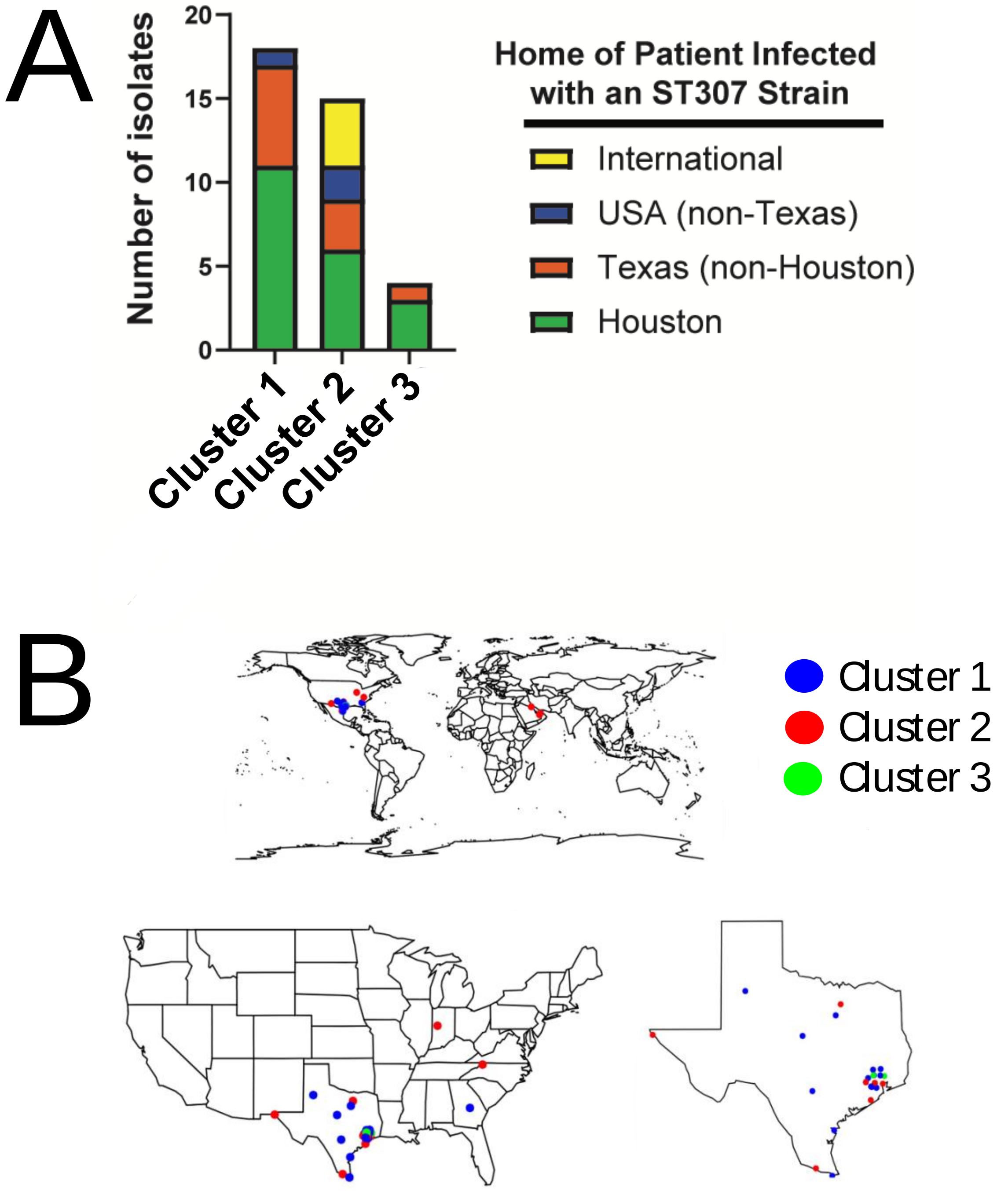

Supplemental Fig. 2. Geographic location of patient origin in which CG307 strains caused bacteremia at our institution. (A) X-axis stratifies isolates by clusters identified using hierarchical clustering. Colors of patient origin as shown in legend. (B) Geographical distribution from a world, country, and state perspective. Isolates are colored by cluster as indicated in legend.

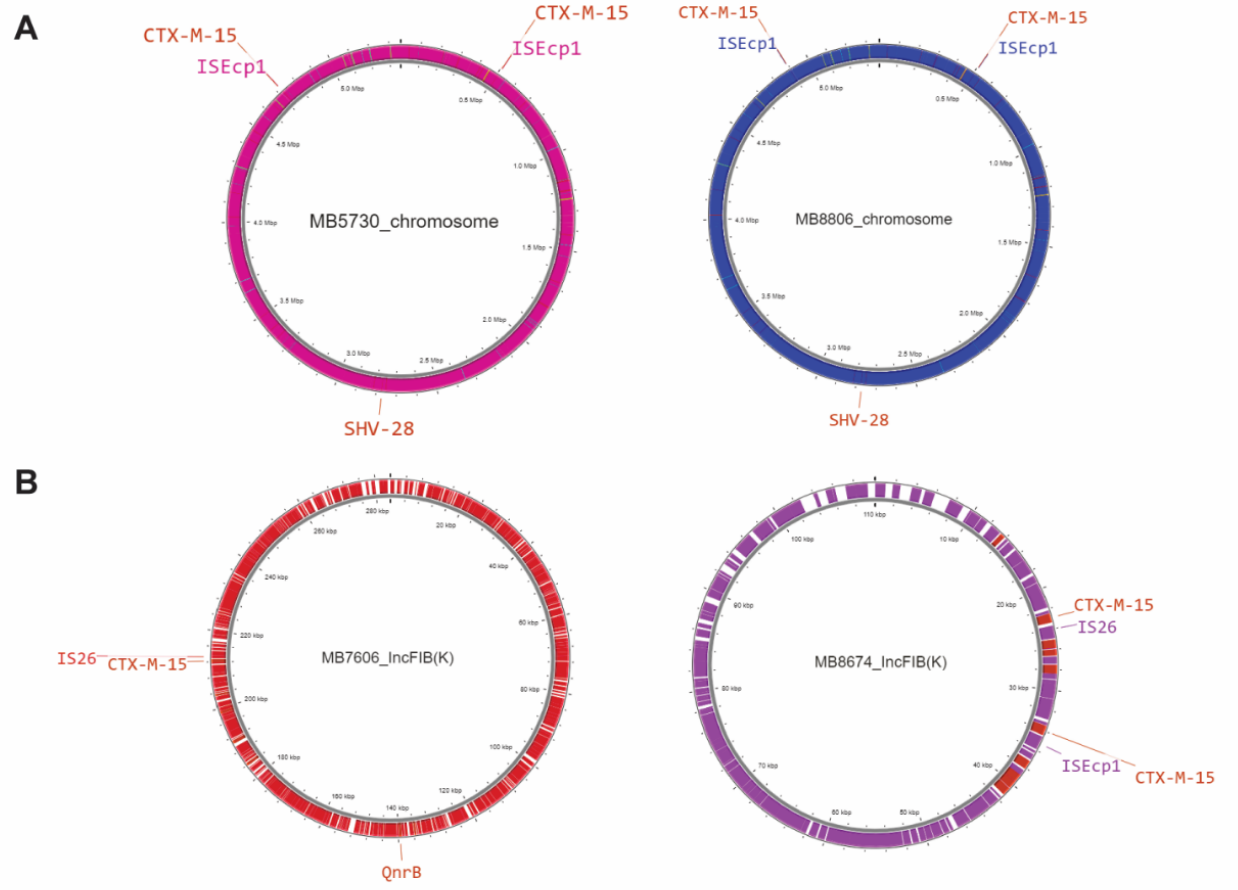

Supplemental Fig. 3. Analysis of CG307 bla$_{CTX\text{-}M\text{-}15}$ copy number and genomic context from MDA 3GC-R*Kp* isolates. (A) CG307 Texas-specific clade isolates (*i.e.,* cluster 3) that have two copies of *bla*$_{CTX\text{-}M\text{-}15}$ present on the chromosome in association with IS*Ecp1* as previously described [49]. (B) CG307 global clade MB7606 isolate (*i.e.,* cluster 2) and novel, US-based clade isolate MB8674 (*i.e.,* cluster 1) that have *bla*$_{CTX\text{-}M\text{-}15}$ present in one or more copies on F-type multireplicon plasmids.
